# Novel Prognostic Signature for Acute Myeloid Leukemia: Bioinformatics Analysis of Combined CNV-Driven and Ferroptosis-Related Genes

**DOI:** 10.3389/fgene.2022.849437

**Published:** 2022-04-26

**Authors:** Chunjiao Han, Jiafeng Zheng, Fangfang Li, Wei Guo, Chunquan Cai

**Affiliations:** ^1^ Clinical School of Paediatrics, Tianjin Medical University, Tianjin, China; ^2^ Department of Pulmonology, Tianjin Children’s Hospital/Tianjin University Children’s Hospital, Tianjin, China; ^3^ Department of Rheumatology and Immunology, Tianjin Children’s Hospital/Tianjin University Children’s Hospital, Tianjin, China; ^4^ Department of Institute of Pediatrics, Tianjin Children’s Hospital/Tianjin University Children’s Hospital, Tianjin, China

**Keywords:** acute myeloid leukemia, copy number variations, ferroptosis, prognosis, gene signature

## Abstract

**Background:** Acute myeloid leukemia (AML), which has a difficult prognosis, is the most common hematologic malignancy. The role of copy number variations (CNVs) and ferroptosis in the tumor process is becoming increasingly prominent. We aimed to identify specific CNV-driven ferroptosis-related genes (FRGs) and establish a prognostic model for AML.

**Methods:** The combined analysis of CNV differential data and differentially expressed genes (DEGs) data from The Cancer Genome Atlas (TCGA) database was performed to identify key CNV-driven FRGs for AML. A risk model was constructed based on univariate and multivariate Cox regression analysis. The Gene Expression Omnibus (GEO) dataset was used to validate the model. Gene Ontology (GO) and Kyoto Encyclopedia of Genes and Genomes (KEGG) enrichment analyses were conducted to clarify the functional roles of DEGs and CNV-driven FRGs.

**Results:** We identified a total of 6828 AML-related DEGs, which were shown to be significantly associated with cell cycle and immune response processes. After a comprehensive analysis of CNVs and corresponding DEGs and FRGs, six CNV-driven FRGs were identified, and functional enrichment analysis indicated that they were involved in oxidative stress, cell death, and inflammatory response processes. Finally, we screened 2 CNV-driven FRGs (DNAJB6 and HSPB1) to develop a prognostic risk model. The overall survival (OS) of patients in the high-risk group was significantly shorter in both the TCGA and GEO (all *p* < 0.05) datasets compared to the low-risk group.

**Conclusion:** A novel signature based on CNV-driven FRGs was established to predict the survival of AML patients and displayed good performance. Our results may provide potential targets and new research ideas for the treatment and early detection of AML.

## Introduction

AML is a heterogeneous malignancy that is characterized by imbalanced hematopoietic stem cells and uncontrolled differentiation. It accounts for 15–20% of leukemia in children and has a higher incidence during adolescence (Arber et al., 2016). Despite intensive treatment, the long-term survival rate of children with AML is still only 45–55% (Carver et al., 2003). Cytogenetic outcomes and molecular is considered the most important prognostic factors at present (Döhner et al., 2017), however, the current genetic methods still can’t achieve accurate prognosis prediction. Our study is aimed to construct a novel prognostic model as well as an improvement of risk-adapted therapy for patients.

Ferroptosis is a newly discovered iron-dependent programmed cell death, which is unlike apoptosis, autophagy, necrosis, pyroptosis, and other cell death forms (Grimwade et al., 2016). It results in cell death by inducing excessive membrane lipid peroxidation (Herold et al., 2018). It is reported that ferroptosis induction can inhibit the growth of tumor cells especially with resistance to traditional therapies (Herold et al., 2014; Döhner et al., 2017). For refractory and relapsed AML, resistance to apoptosis is an important therapeutic measure (Hong et al., 2021; Huang et al., 2021). Currently, as a potential therapeutic target for cancer treatment, ferroptosis has attracted worldwide attention. Some studies have suggested that some ferroptosis-related genes can be prognosis biomarkers (Huot et al., 1996; Herold et al., 2014; Döhner et al., 2017). The upregulation of phospholipid hydroperoxidase GPx4 is associated with poor prognosis of AML (Jakob et al., 1993). In addition, a study found that low AKR1C2 and SOCS1 expression was highly correlated with more favorable overall survival and disease-free survival in AML patients (Jiang et al., 2020).

CNV refers to the duplication, inversion, or deletion of a DNA sequence of more than one kilobase (Jiang et al., 2020). Recently, CNV has been recognized as an important source of genetic variation which was found to play an important role in many cancers. The previous study suggested that the presence of CNV affects not only protein expression but also long non-coding RNAs and miRNAs (Kerr et al., 1994). Cytogenetic CNV abnormalities have been included in WHO classification (2016) (Kim et al., 2012) and other risk stratification strategies (Liang et al., 2009; Li et al., 2013; Kuett et al., 2015), and constitute the single strongest prognostic factor for complete remission (CR) and overall survival (OS) of AML. A study has found CNVs in 23 patients (76.7%) with NK-AML in Korea (Jiang et al., 2020). It showed that CNV increase is an independent predictive factor for shorter event-free survival and may affect the success of Ara-C and anthracycline-based chemotherapy. However, most previous studies focused on CNV or transcriptome changes, there is still a lack of comprehensive research on how CNV drives AML.

Although FRGs and CNV can be used as prognostic markers of AML respectively, the relationship between FRGs and CNV has not been reported at present, which aroused our interest. In the present study, we used transcriptomics and CNVs profiles to identify CNV driven FRGs and aimed to construct a prognostic model of AML. Our study may contribute to a better understanding of the underlying mechanisms and provide a new therapeutic target for the treatment of AML.

## Materials and Methods

### Data Source

Gene expression profiles of 151 bone marrow samples from AML patients were downloaded from the TCGA database. Since sequencing data of control samples were not available from the TCGA database, we obtained RNA sequencing data of 337 normal peripheral blood samples from the GTEx database. The GSE37642 (Mitra et al., 2008; Liang et al., 2016; Meng et al., 2016; Nibourel et al., 2017) was obtained from the GEO database. The GSE37642 dataset (platform: GPL570; https://www.ncbi.nlm.nih.gov/geo/query/acc.cgi?acc=GSE37642) contains transcriptional data from 136 AML patients with complete survival information, which was used for external validation analysis of the prognostic signature. Furthermore, the GSE12417 (platform: GPL570; n = 73; https://www.ncbi.nlm.nih.gov/geo/query/acc.cgi?acc=GSE12417) and GSE71014 (platform: GPL10558; n = 104; https://www.ncbi.nlm.nih.gov/geo/query/acc.cgi?acc=GSE71014) datasets were downloaded as complementary external validation sets to perform validation analyses on a larger sample base.

**FIGURE 1:**
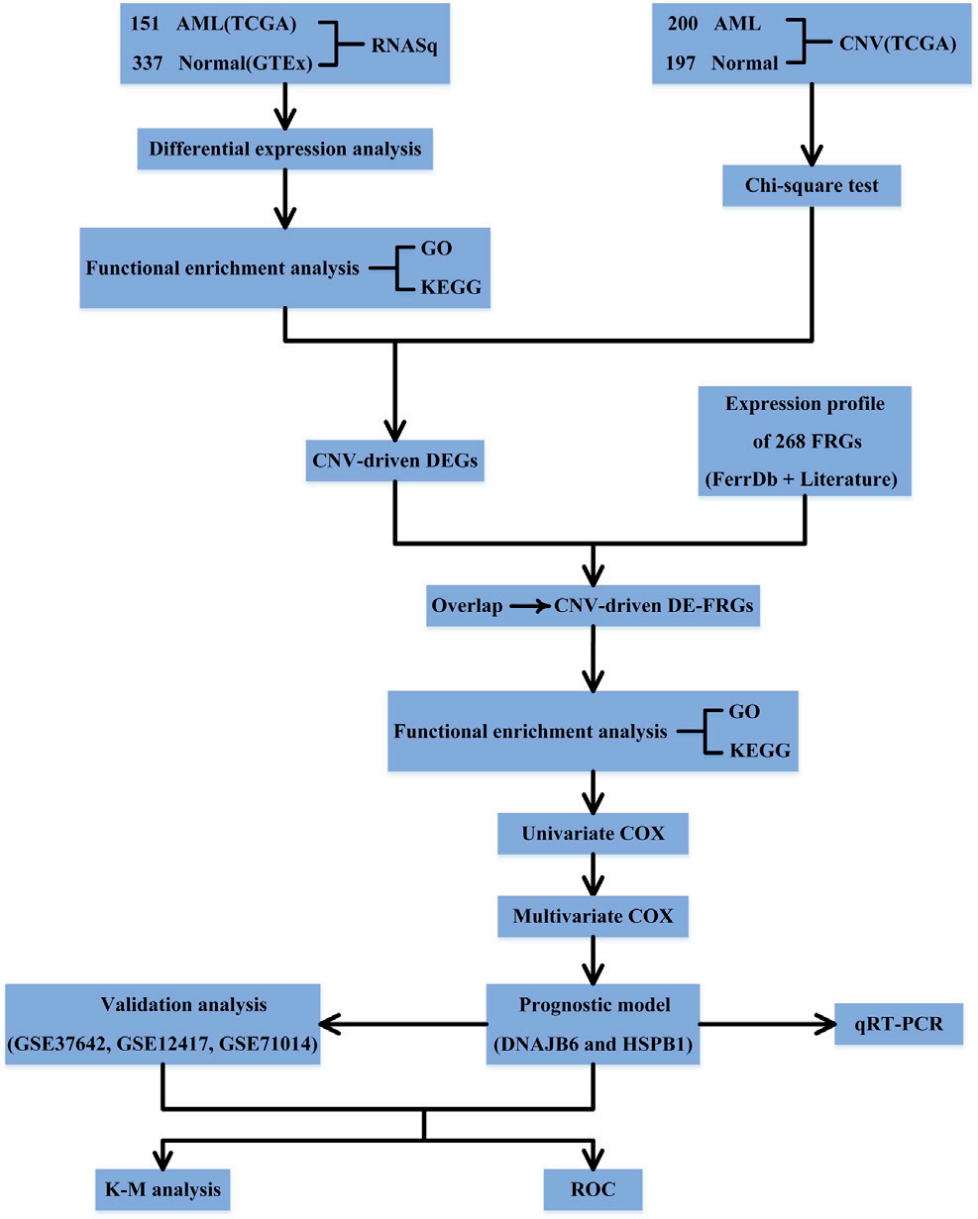
Schematic presentation of this study.

Figure 1 shows the schematic presentation of this study.

### Collection of FRGs

We downloaded 259 FRGs from the FerrDb online database (http://www.zhounan.org/ferrdb/). Meanwhile, a total of 60 FRGs were also obtained from the report of Yingkai Hong et al. (Herold et al., 2014). After de-duplication (retention of unique values), a total of 268 FRGs were obtained for our study (Supplementary Table S1).

### Differential Expression Analysis

Gene expression profiles of AML (n = 151) and normal (n = 337) samples from TCGA and GTEx were subjected to normalize Between Arrays function for normalization, and subsequently, differential expression analysis (AML vs. normal) was performed using the R package limma. The significance threshold was set to |log_2_ fold change (FC)| ≥ 1 and *p* < 0.05. A volcano map showing the distribution of the identified DEGs was plotted based on the R package ggplot2. In addition, we also verified that the identified DEGs were able to distinguish between normal and AML samples by Principal Component Analysis (PCA) to imply the applicability of the samples.

### Functional Enrichment Analysis

To reveal the functions of target genes, R package clusterProfiler (Papaemmanuil et al., 2016) was used to conduct Gene Ontology (GO) annotation and Kyoto Encyclopedia of Genes and Genomes (KEGG) pathway enrichment analyses. The GO terms were comprised of the following three divisions: biological process (BP), cellular component (CC), and molecular function (MF). *p* < 0.05 was regarded as statistically significant.

### Integrative Analysis of Gene Expression and CNVs

The CNV data of AML patients (n = 200) and normal subjects (n = 197) were downloaded from the TCGA database (sequencing platform Affymetrix SNP 6.0). Genes in CNV regions were annotated using Genome Research Consortium Human build 38 (GRCh38) as the reference genome. CNVs alteration rates between normal and tumor samples were then compared using the Chi-square test, and CNVs data with *p* < 0.05 were chosen for the next analysis. Then the CNVs data and DEGs data of the same sample were merged to construct a matrix. By using the Kolmogorov-Smirnov test, those genes showing the same tendency both in CNVs and differential gene expression (CNV increase-upregulated; CNV decrease-downregulated) were selected as CNV-driven DEGs. Moreover, CNV-driven DEGs and identified FRGs were analyzed for overlap, and the common genes in both gene lists were defined as CNV-driven DE-FRGs.

### Construction, Evaluation, and Validation of the Prognostic Model

The TCGA-AML dataset containing 132 AML samples with complete survival information was used for prognostic gene screening and prognostic model construction and evaluation. The GSE37642 (n = 136), GSE12417 (n = 73), and GSE71014 (n = 104) datasets were used as an independent external validation set for prognostic model validation analysis. Prognostic genes were screened by Cox regression analysis. Briefly, target genes were included in univariate Cox regression analysis, and variables satisfying *p* < 0.1 were included in stepwise regression multivariate Cox analysis. The variables obtained from multivariate Cox regression analysis were identified as the optimal variables for the construction of the prognostic model. The risk score for each AML patient was calculated using the regression coefficient (coef) calculated from the multivariate Cox analysis and the expression of prognostic genes. The formula for calculating the risk score as shown below:

$$risk\;score=coef_{gene_{1}}\times expression_{gene_{1}}+coef_{gene_{2}}\times expression_{gene_{2}}+\cdots +coef_{gene_{n}}\times expression_{gene_{n}}$$

The samples were classified into high- and low-risk groups based on the median value of the risk score in each dataset (TCGA dataset and independent external validation set). The difference in OS between the two risk subgroups was assessed based on the R package survival using Kaplan-Meier (K-M) analysis. Receiver operating characteristic (ROC) curves were plotted by survROC package to assess the accuracy of the risk score for prognostic prediction in patients with TCGA-AML and GSE37642/GSE12417/GSE71014 datasets-AML.

### Independent Prognostic Analysis of the Risk Score

Clinical characteristics of AML (age and sex) available in the TCGA database were included in the Cox regression analysis along with the risk score. Univariate Cox regression analyses with *p* < 0.05 output would be performed further in multivariate Cox analyses. Ultimately, variables with *p* < 0.05 generated by multivariate Cox regression analysis were considered as independent prognostic factors for AML.

### Patient and Tissue Preparation

We selected the blood of 10 AML patients and 10 healthy people to carry on the quantitative PCR test to the genes screened in this study. The experimental verification data are obtained from the sample database of the Institute of Pediatrics of Tianjin Children’s Hospital, and no samples are obtained directly from the children’s body, so they do not need to be approved by the institutional ethics committee. All experimental operations are carried out in accordance with the relevant guidelines and regulations, and have been repeatedly verified by professional laboratory researchers.

### RNA Isolation and Quantitative Real-Time Polymerase Chain Reaction

Whole-cell RNA was extracted via the RNAiso Plus (TaKaRa, Japan). Quantitative real-time polymerase chain reaction (qRT-PCR) was performed to detect DNAJB6 and HSPB1 expression using American Bio rad Bole T100 gradient PCR instrument. The following primers were used for qRT-PCR: DNAJB6 primers: upstream primer F: 5′-TAT​GAA​GTG​CTG​TCG​GAT​GCT​AAG​A-3'; downstream primer R: 5′-GAA​GAC​ATC​ATC​TGG​GTT​ACG​GA-3'. The conditions for PCR to DNAJB6 were: the size of the amplified product was 144bp, annealing temperature was 55–60°C. The sequences of HSPB1 primers used were: upstream primer F: 5′-GCA​GTC​CAA​CGA​GAT​CAC​CA-3′ and downstream primer R: 5′-TTA​CTT​GGC​GGC​AGT​CTC​ATC-3′. The conditions for PCR to HSPB1 were: the size of the amplified product was 97bp, annealing temperature was 55–60°C. qRT-PCR was conducted in triplicate for each sample. All gene expression levels were normalized to that of GAPDH using the 2^−ΔΔCt^ method. Uupaired *t*-test (two-tailed) was used for the comparison analyses.

### Statistical Analysis

All analyses in this study were performed in R software. A log-rank test was used to check the significant difference in OS between groups. An area under the ROC curve (AUC) served as an indicator of prognostic accuracy. Unless otherwise specified, a *p*-value less than 0.05 was considered statistically significant.

## Results

### Exploration of AML-Related DEGs

**FIGURE 2:**
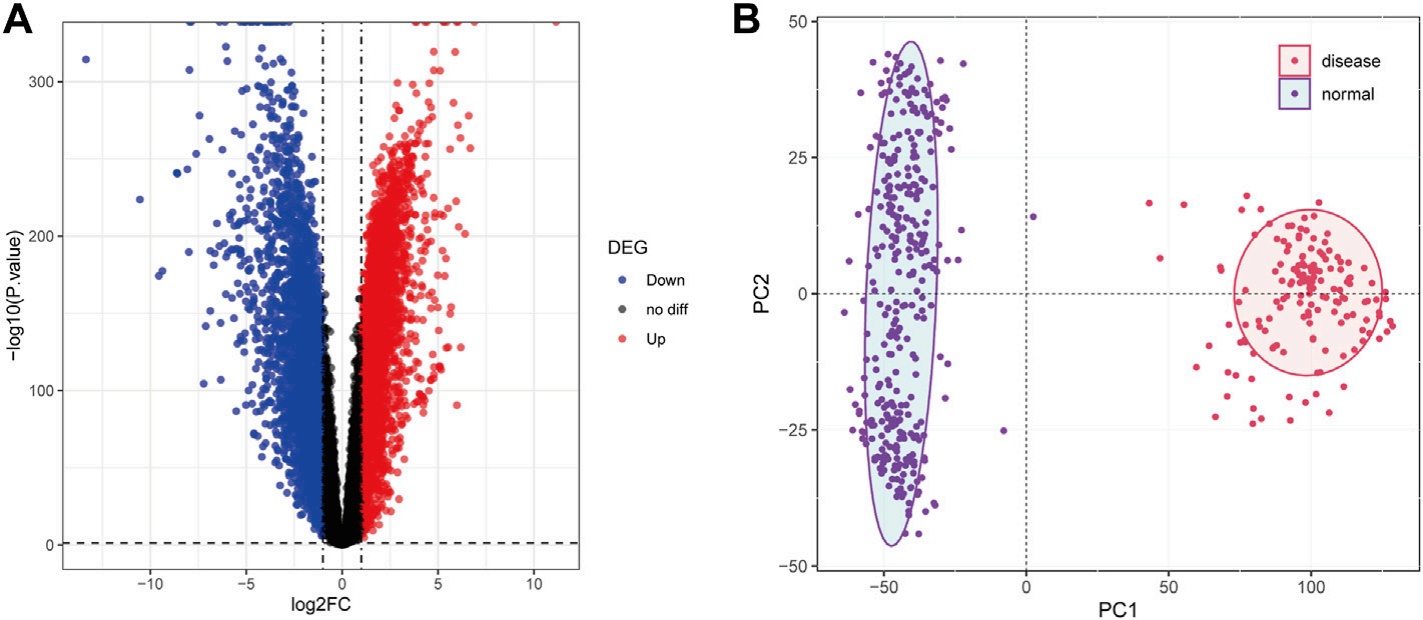
Differential expression analysis. **(A)**: The volcanic map was used to show the differential genes between the samples of AML patients and normal people. Abscissa denotes log2FC, ordinate denotes-log10 (*p* value). Each dot in the picture represents a gene, and the red and blue dots represent significant differential expression, and the red dots indicate upregulated expression in the disease samples, blue dots indicate downregulation, and black dots indicate no significant difference. The horizontal guide represents-log10 (*p*-value) = 0.05. The vertical guide represents log2FC = ±1. **(B)**: Principal component analysis diagram. The dots in the picture represent the sample, the red represents the AML patient, and the purple represents the normal person sample. PC1 and PC2 represent the first and second principal components respectively.

The normalized expression profiles of TCGA-AML (n = 151) and GTEx-normal (n = 337) were selected as the basis for differential expression analysis. By R package limma, we identified 6,828 DEGs between AML and normal samples. Among them, a total of 3,330 met log_2_ FC ≥ 1 and *p* < 0.05 and 3,498 matched log_2_ FC ≤ -1 and *p* < 0.05 (Figure 2A; Supplementary Table S2). Furthermore, PCA performed based on the obtained DEGs demonstrated that samples from different groups were clustered in the same category (Figure 2B).

**FIGURE 3:**
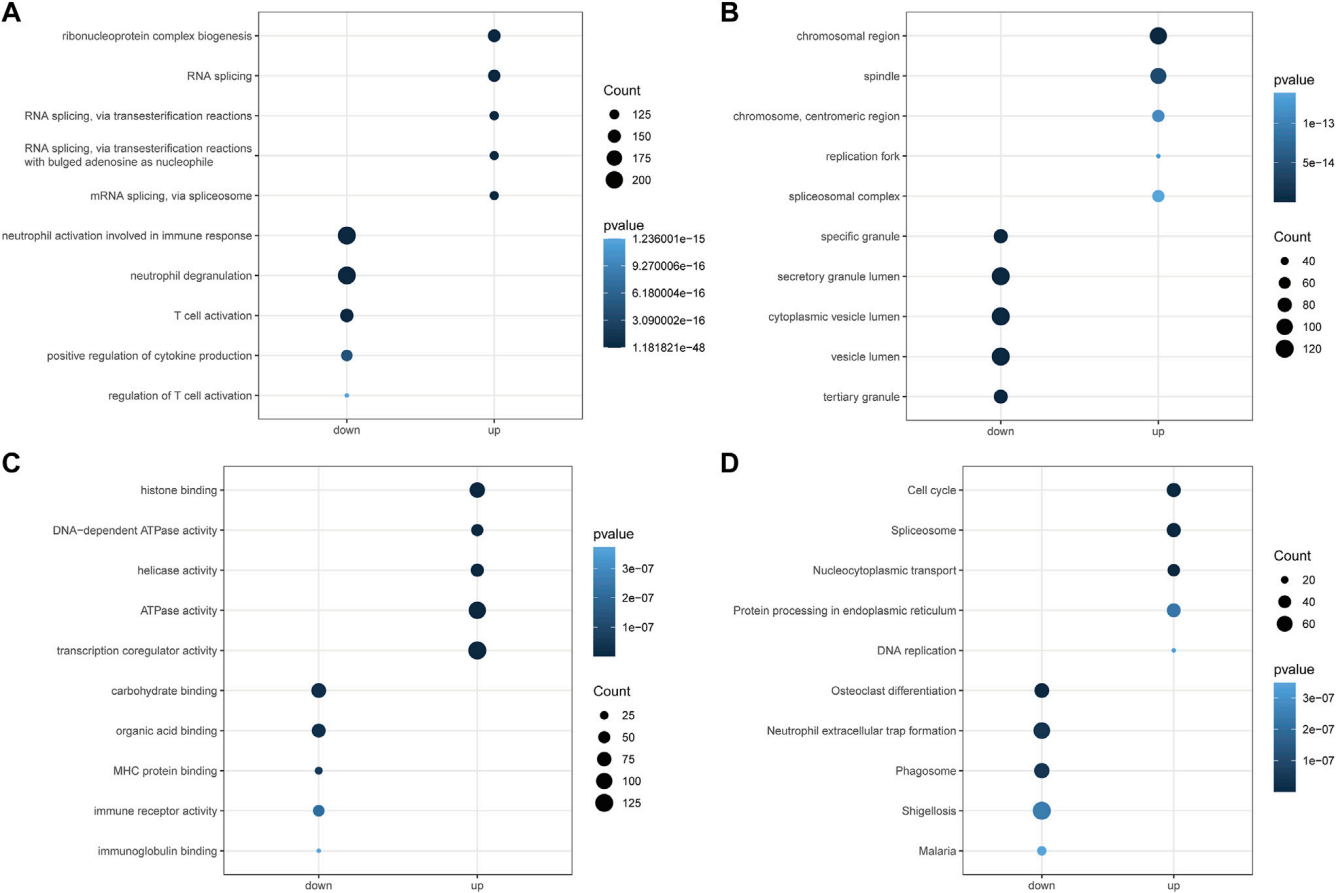
Functional enrichment analysis of differential genes by GO and KEGG. **(A)**: Gene ontology analysis for the biological process (BP) of the AML-related DEGs. **(B)**: Gene ontology analysis for the cellular component (CC) of the AML-related DEGs. **(C)**: Gene ontology analysis for the molecular function (MF) of the AML-related DEGs. **(D)**: Kyoto Encyclopedia of Genome and Genome (KEGG) enrichment analysis of the 6828 AML-related DEGs. Down represents the functional enrichment of downregulated genes, while up represents the enrichment analysis of up-regulated genes. The vertical axis represents the functional item, the size of the dot represents the number of enriched genes, and the color represents the *p*-value.

To explore the potential mechanisms of the above AML-related DEGs, we performed GO and KEGG function enrichment analysis for upregulated DEGs and downregulated DEGs, respectively, using the clusterProfiler package. Figures 3A–C illustrated the top5 terms that were significantly enriched in the three categories of GO, BP, CC, and MF, based on upregulated and downregulated DEGs. In the BP category, specifically, upregulated DEGs were significantly associated with “ribonucleoprotein complex biogenesis”, “RNA splicing”, “RNA splicing, via transesterification reactions” (Figure 3A); besides, these genes were found to be closely correlated with the cell cycle (“DNA replication”, “chromosome segregation”, “mitotic nuclear division”, etc.). Moreover, upregulated DEGs might be played mainly in CCs such as “chromosomal region”, “spindle”, “chromosome, centromeric region” **(** Figure 3B) for MFs such as “histone binding”, “DNA-dependent ATPase activity”, “helicase activity” (Figure 3C). The results of the detailed GO analysis for the upregulated DEGs could be reviewed in Supplementary Table S3. For the down-regulated DEGs, they were significantly correlated with immune responses (“regulation of immune effector process”, “negative regulation of immune system process”, “immune response-activating signal transduction”, etc.) and biological processes of immune cells (“neutrophil degranulation”, “T cell activation”, “T-cell differentiation”, “lymphocyte proliferation”, etc.) (Figure 3A). Also, these genes were able to perform the molecular functions of “carbohydrate binding”, “organic acid binding”, and “MHC protein binding” (Figure 3C) in “specific granule”, “secretory granule lumen”, and “cytoplasmic vesicle lumen” (Figure 3B). Detailed GO analysis results for downregulated DEGs were displayed in Supplementary Table S4. The top5 pathways significantly enriched in KEGG analysis were shown in Figure 3D, with upregulated DEGs involved in “Cell cycle”, “Spliceosome”, “Nucleocytoplasmic transport”, “Protein processing in endoplasmic reticulum”, and “DNA replication” (Supplementary Table S5); downregulated DEGs were closely associated with “Osteoclast differentiation”, “Neutrophil extracellular trap formation”, “Phagosome”, “Shigellosis”, and “Malaria” (Supplementary Table S6).

### Identification of CNV-Driven DE-FRGs in AML Patients

**FIGURE 4:**
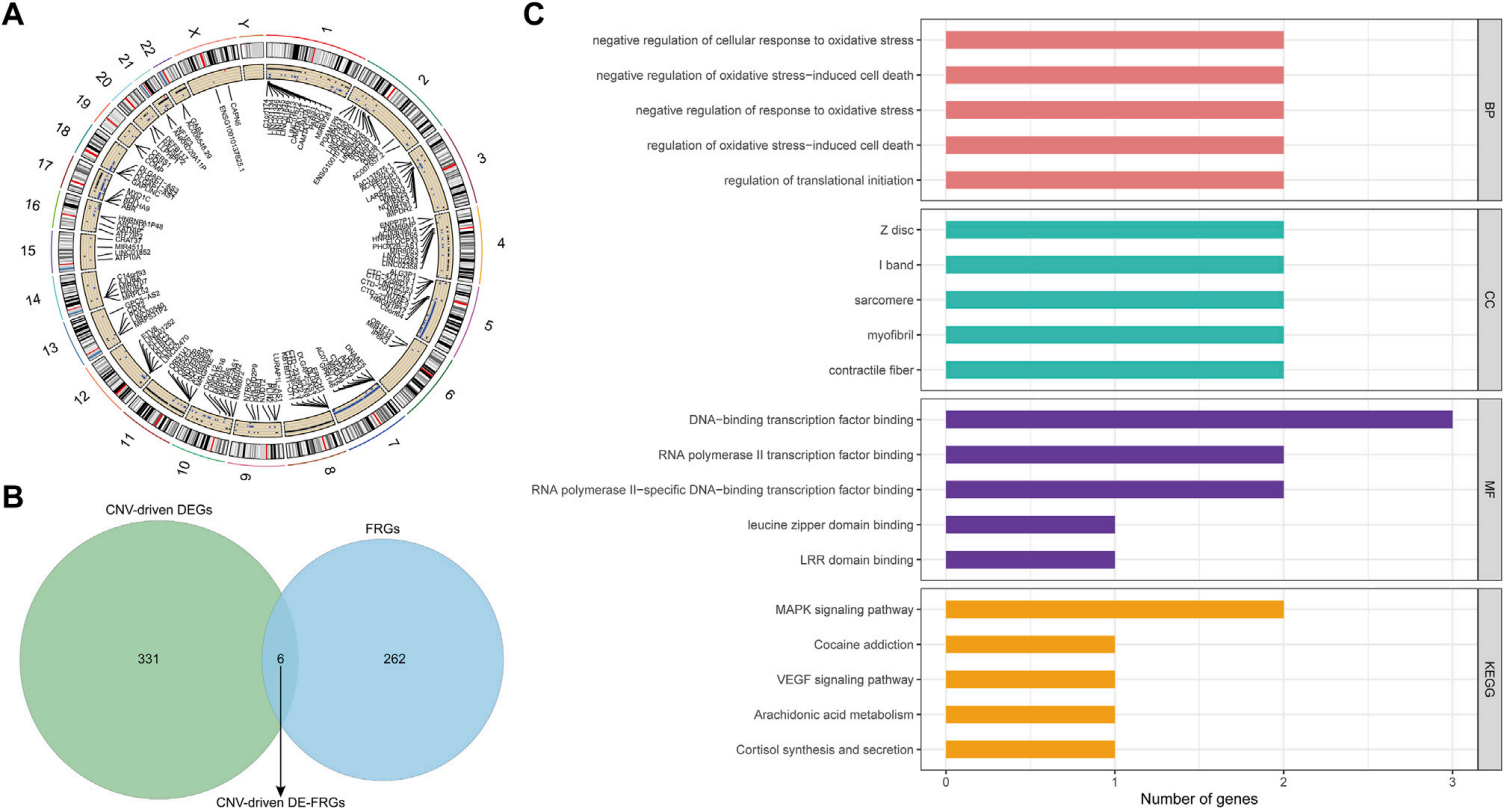
Identification and function analysis of the CNV-driven differential ferroptosis-related genes in AML. **(A)**: Distribution of AML-related CNVs visualized by circus plot. The outside circle represents 24 chromosomes including sex chromosomes; the inside circle represents the distribution of CNVs (the blue dots represent CNV deletions). **(B)**: ferroptosis-related genes and CNV driving genes were intersected and Venn diagram was drawn. Finally, six differential ferroptosis-related genes driven by CNV were obtained: ALOX15B, MTDH, DNAJB6, HSPB1, ATF4, and PLIN2. **(C)**: The functions of 6 CNV-driven differential ferroptosis-related genes were analyzed by GO and KEGG functional enrichment analysis. The first five functional items were sorted according to the count value, the horizontal axis represented the number of enriched genes, and the vertical axis represented the functional items.

By applying the Chi-square test, 4637 CNV genes associated with AML were identified (*p* < 0.05; Supplementary Table S7). The distribution of AML-related CNVs in chromosomes was presented in Figure 4A. Then CNV-driven DEGs were screened using the Kolmogorov-Smirnov test. We selected 337 CNV-driven DEGs, of which 127 were upregulated in AML with increased CNV (Supplementary Table S8) and the remaining 210 were downregulated with decreased CNV (Supplementary Table S9). Subsequently, by overlap analysis (Figure 4B), a total of 6 CNV-driven DE-FRGs, namely ALOX15B, MTDH, DNAJB6, HSPB1, ATF4, and PLIN2, were identified in the above list of CNV-driven DEGs and the list of 268 FRGs (Supplementary Table S1).

To illustrate the potential functional and biological effects of these CNV-driven DE-FRGs, GO (Supplementary Table S10) and KEGG (Supplementary Table S11) analyses were performed (Figure 4C). Results showed that CNV-driven DE-FRGs were significantly enriched in the BP category in terms related to oxidative stress response and its mediated cell death, such as “negative regulation of cellular response to oxidative stress”, “negative regulation of oxidative stress-induced cell death”, “negative regulation of response to oxidative stress”, and “regulation of oxidative stress-induced cell death”. KEGG analysis showed that these genes were involved in “MAPK signaling pathway”, “Cocaine addiction”, “VEGF signaling pathway”, “Arachidonic acid metabolism”, and “Cortisol synthesis and secretion”. These results suggested that CNV-driven DE-FRGs were probably implicated in the cell cycle dysregulation and the inflammatory immune response during the disease process.

### Construction of a Prognostic Signature Based on CNV-Driven DE-FRGs

**FIGURE 5:**
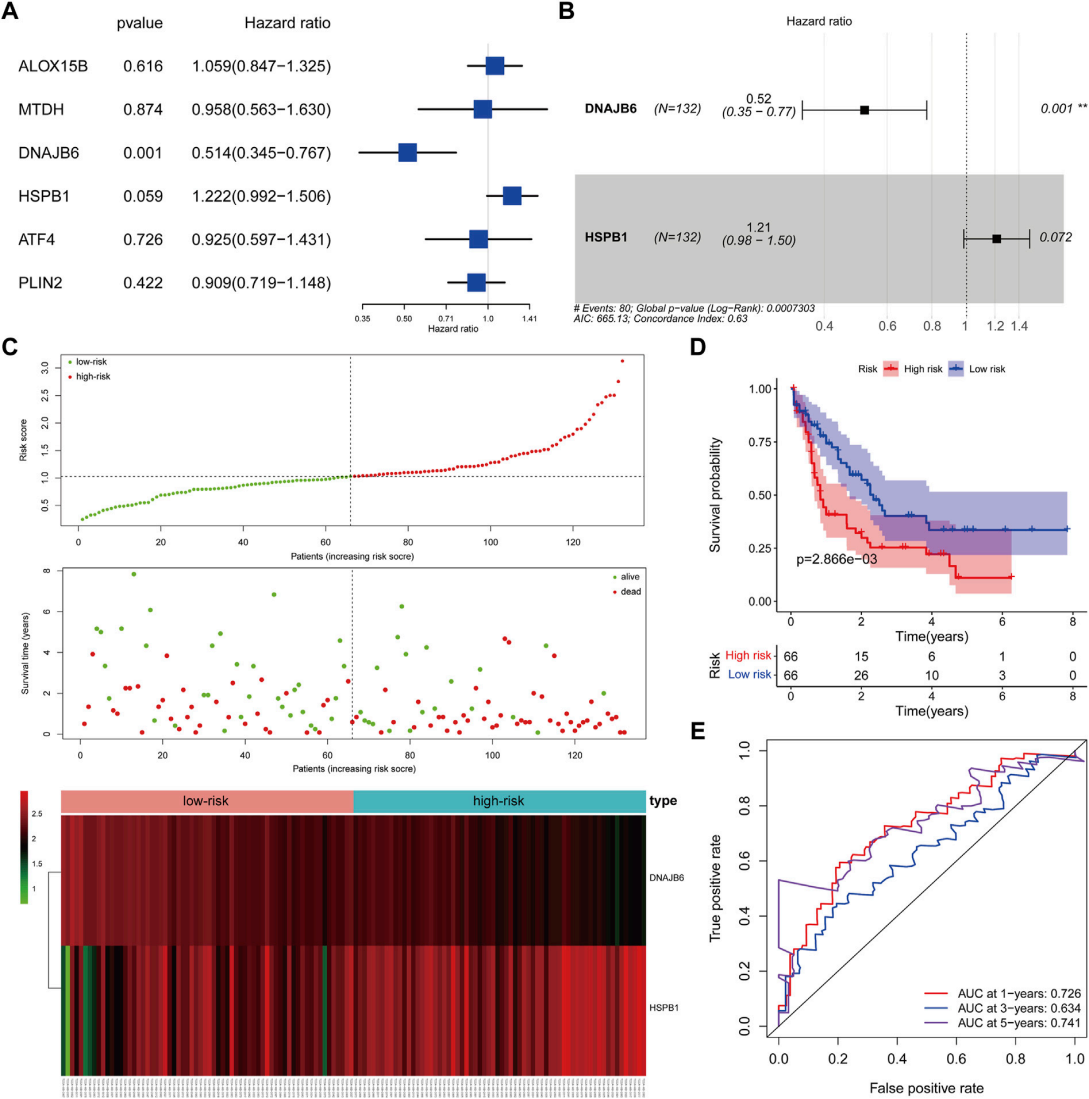
Clinical prognostic biomarker analysis. **(A)**: Forest map shows univariate Cox analysis. **(B)**: forest map shows multivariate Cox analysis, Hazard ratio >1, high-risk gene, that is, the higher the gene expression, the higher the patient risk; conversely, Hazard ratio <1, low-risk gene, that is, the lower the gene expression, the higher the patient risk. **(C)**: The risk model draws a risk curve, with red dots representing high-risk patients and green dots representing low-risk patients. The ordinate is the risk score and the survival time respectively, and the dotted line is the median risk score and the corresponding number of diseases. Gene expression heatmap of high-risk and low-risk groups. **(D)**: According to the K-M survival curve drawn by the risk score, the Abscissa represents the survival time, the ordinate indicates the survival rate, the red curve represents the high-risk group, and the blue curve represents the low-risk group. **(E)**: ROC curve (1, 3, 5 years), the AUC area of the model was calculated to evaluate the effectiveness of the model.

We matched the expression profiles of the identified CNV-driven DE-FRGs in 132 TCGA-AML samples containing complete survival data. Univariate Cox regression analysis was applied to identify the CNV-driven DE-FRGs associated with survival time in AML patients. *p* < 0.1 was set as the cut-off value, and a total of 2 variables associated with survival in TCGA-AML patients were screened, namely DNAJB6 and HSPB1 (Figure 5A). HR > 1 for HSPB1 (HR = 1.2222, 95% CI. 0.992–1.506, *p* = 0.059) may be an oncogenic gene in AML, whereas DNAJB6 (HR = 0.514, 95% CI: 0.345–0.767, *p* = 0.001) with HR < 1 was expected to be a protective factor for AML. Further, the Cox model consisting of DNAJB6 and HSPB1 (Figure 5B) was identified as the optimal prognostic signature for AML by sophisticated calculations of multivariate Cox analysis with stepwise regression.

### Evaluation of a Two-Gene Prognostic Signature-based Risk System

We assessed the efficacy of prognostic signature consisting of DNAJB6 and HSPB1 for predicting AML prognosis by risk scoring system. The risk score for each TCGA-AML patient was calculated according to the following formula: risk score = (−0.65 * expression of DNAJB6) + (0.19 * expression of HSPB1). All TCGA-AML samples were divided into high- (n = 66) and low- (n = 66) risk groups according to the median risk score (median = 1.029) (Supplementary Table S12). Figure 5C demonstrated the risk profile and AML survival distribution in the TCGA database, suggesting that low-risk AML patients had relatively longer OS than high-risk patients. Moreover, the heatmap showed that DNAJB6 was relatively less expressed in the high-risk group compared to the low-risk group, while HSPB1 tended to be more highly expressed in the high-risk group (Figure 5C). K-M survival analysis confirmed that the low-risk group had better OS and the high-risk score was linked to poor outcomes (Figure 5D). The ROC curve assessed the accuracy of the risk score to predict OS in TCGA-AML patients at 1, 3, and 5 years 1 year OS had an AUC of 0.726, 3 years of 0.634, and 5 years of 0.741 (Figure 5E). These results indicated that our risk score had a more reliable performance in predicting the prognosis of AML patients.

### Validation of the Two-Gene Prognostic Signature in the GEO Database

**FIGURE 6:**
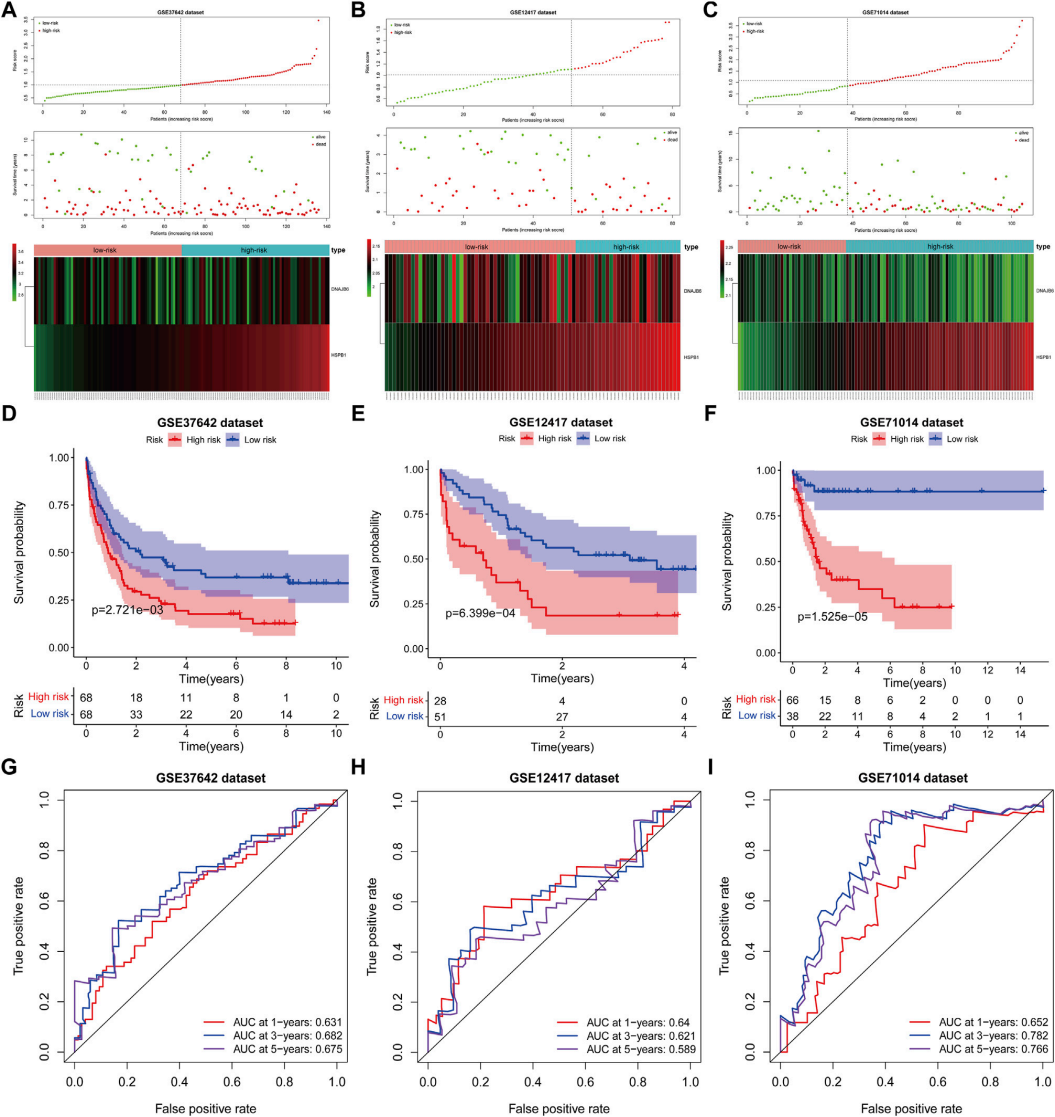
The prognostic risk model was validated in the GEO database. **(A–C)**: The distribution of risk score, survival time, life status, and the prognostic 2-CNV-driven DE-FRGs expression patterns in the GSE37642 dataset, GSE12417 dataset and GSE71014 dataset. The risk scores are arranged in ascending order from left to right and each dot indicates an AML individual. The black dotted line is the optimum cutoff dividing patients into low and high-risk groups. The colors from green to red in the heatmap indicate the expression level from low to high. **(D–F)**: Kaplan-Meier plots compare overall survival between patients in low- and high-risk groups in the GSE37642 dataset, GSE12417 dataset and GSE71014 dataset. *p*-values were calculated by log-rank test. **(G–I)**: The receiver operating characteristic (ROC) curves of the prognostic signature for 1-, 3-, and 5-years survival in the GSE37642 dataset, GSE12417 dataset and GSE71014 dataset.

The risk scores for AML patients in the GSE37642 (n = 136), GSE12417 (n = 73), and GSE71014 (n = 104) datasets were calculated using the above equations, and the samples were divided into high-risk and low-risk groups based on the optimal threshold for risk scores (Figures 6A–C). The predictive validity of the risk scores in the independent external validation set was consistent with that in TCGA database. Patients in the low-risk group had significantly longer survival times compared with the high-risk group (Figures 6D–F). Meanwhile, ROC curve analysis showed that the AUC of risk score in predicting patients” OS at 1, 3, and 5 years was 0.631, 0.682, and 0.675 in the GSE37642 dataset (Figure 6G). 0.640, 0.621, and 0.589, respectively, in the GSE12417 dataset (Figure 6H). 0.652, 0.782, and 0.766, respectively, in the GSE71014 dataset (Figure 6I). This evidence suggested that our risk score had more satisfactory predictive validity and some general applicability.

### Risk Score was an Independent Prognostic Factor for AML

We explored the relationship between risk score and clinical characteristics (age and gender) in the TCGA-AML dataset by the Wilcoxon test. The results revealed that the risk score was significantly different between age subgroups (*p* = 0.02), and the risk score was positively correlated with age, with higher risk levels at older ages (Supplementary Figure 1A). Besides, the risk scores were higher in the male subgroup than in the female group, but which was not statistically significant (*p* = 0.084; Supplementary Figure 1B).

Further, Cox regression analysis was utilized to evaluate whether the risk score could predict the outcome of AML patients independently of clinical characteristics (age and gender). Univariate Cox analysis noted that age and risk score were significantly associated with prognosis in AML patients (*p* < 0.05; Table 1). Ultimately, multivariate Cox analysis indicated that age and risk score were the independent factors of AML prognosis (Table 2).

**TABLE 1:** Univariate Cox analysis.

	HR	HR.95L	HR.95H	*p*-value
Age	1.03482198014829	1.01882519224958	1.05106993696686	1.66E-05
Gender	0.962366615022004	0.619557528552139	1.49485634348315	0.864443099593092
Risk score	2.55281810175952	1.70387284566633	3.82474565355407	5.54E-06

**TABLE 2:** Multivariate Cox analysis.

	HR	HR.95L	HR.95H	*p*-value
Age	1.03132276756969	1.01480193820568	1.04811255365579	0.000181619864716146
Risk score	2.26714423194939	1.47977186168049	3.47346986489139	0.000169704374503001

### Validation of Prognostic Gene Expression in PCR Expression Test

**FIGURE 7:**
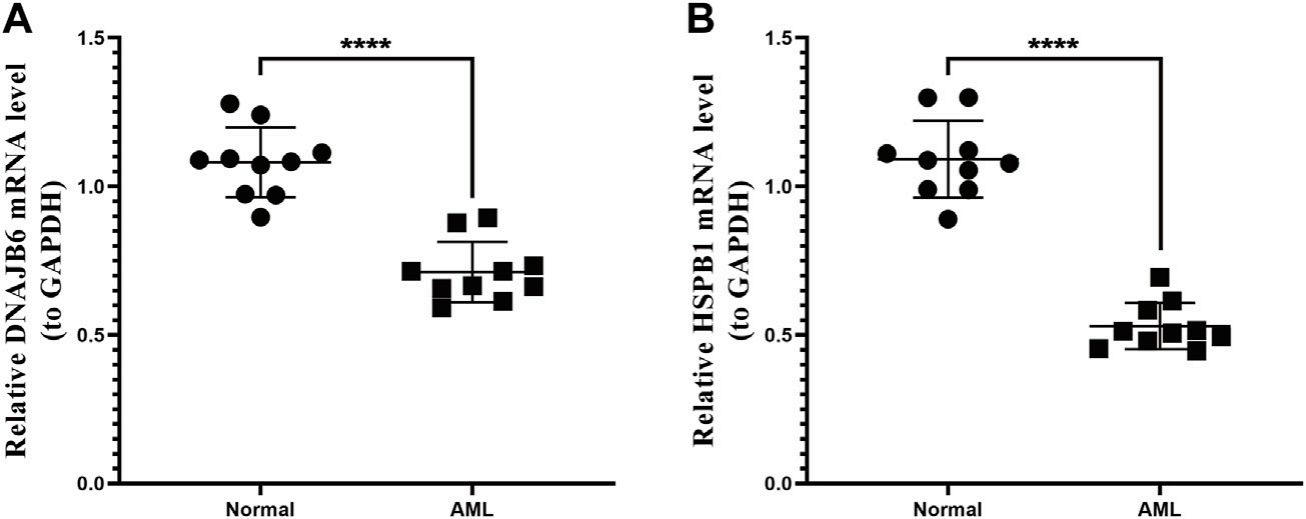
qRT-PCR expression test. The two genes selected in the model were verified by qRT-PCR expression test. **(A)**: Expression of DNAJB6 gene in normal human and AML patients. **(B)**: Expression of HSPB1 gene in normal human and AML patients. *p*-values were calculated by the independent sample *t*-test.

We collected the blood of 10 AML patients and 10 healthy individuals and analyzed the mRNA expression levels of two prognostic genes by PCR. The results showed that the expression of the DNAJB6 gene and the HSPB1 gene in AML patients were both lower than that in healthy people (*p* < 0.0001; Figures 7A,P < 0.0001; Figure 7B). There was a significant statistical difference in the expression level after statistical analysis.

## Discussion

With the progress of AML treatment such as the combination of chemotherapy and stem cell transplantation, the outcomes of AML patients have great improvement. However, the prognosis of AML patients still cannot be accurately judged. The defect of apoptosis is a common cause of chemoresistance (Poeta et al., 2008). Ferroptosis is different from apoptosis which can provide us with new ideas for inducing cancer cell death (Radtke et al., 2009). Some reports have determined that leukemia cells are more sensitive to the ferroptosis inducer erastin than other cancer cell types (Seth and Singh, 2015; Reyna et al., 2017). In addition, CNV has been reported to be associated with chemotherapy response and many studies on CNV in AML have been carried out (Song et al., 2016; Shen et al., 2018; Jiang et al., 2020; Shao et al., 2021). However, none of them has comprehensively evaluated the role of gene expression derived from CNV. Both ferroptosis and CNV can be used as prognostic markers, but the relationship between them in AML has not been reported. Therefore, we combined ferroptosis and CNV aiming to improve the prognostic prediction and management efficiency in AML patients.

We combined analysis of CNV differential data and differentially expressed genes (DEGs) data to identify key CNV-driven FRGs for AML by using publicly available AML datasets. A total of 6 CNV-driven DE-FRGs (ALOX15B, MTDH, DNAJB6, HSPB1, ATF4, and PLIN2) were identified by overlap analysis, and functional enrichment analysis indicated that they were involved in oxidative stress, cell death, and inflammatory response processes. Finally, 2 CNV-driven FRGs (DNAJB6 and HSPB1) were identified by Univariate analysis and COX model. The OS of patients in the high-risk group was significantly shorter in the datasets compared to the low-risk group.

DNAJB6 encodes a highly conserved DNAJ/Hsp40 family chaperone protein and interacts with Hsp70 chaperone protein (Stevens et al., 2019). Glutathione peroxidase 4 (GPX4, an antioxidant enzyme) is a defensive protein which is a kind of the GSH peroxidase (Sun et al., 2015). GPx4 inhibits lipid reactive oxygen species (ROS) production by decreasing phospholipid hydroperoxide and plays an important role in inhibiting iron cell apoptosis (Reyna et al., 2017). Overexpressing DNAJB6a can has the ability to downregulate GPX4 and promote ferroptosis (Tang et al., 2011). It is reported that DNAJB6 expression was downregulated in ESCC tissues and it acts as an anti-oncogene in ESCC (Tang et al., 2011). In addition, Mitra A et al. also reported that DNAJB6a can weaken malignant activity of breast carcinoma (Vosberg et al., 2016). However, Zhang et al. found that DNAJB6 is an oncogene that can aggravate the invasion of colorectal cancer (Wei et al., 2020). In our study, we found the expression of DNAJB6 oncogene was lower in the low-risk group compared with the high-risk group associated with poor prognosis. At the same time, according to the hazard ratio (HR) of univariate analysis, the HR of DNAJB6 is 0.514, indicating that DNAJB6 is a protective factor in AML. Our result is consistent with previous study about ESCC and breast cancer.

HSPB1 (also named Hsp27), a member of the small heat shock protein family, is involved in regulating cytoskeletal tissue or stabilizing abnormally folded proteins to prevent aggregation (Yang et al., 2013; Yan et al., 2021). Its abnormal expression in cancer is associated with aggressive tumor behavior, increased chemoresistance, and poor prognosis (Carver et al., 2003). It is overexpressed in many cancers such as prostate, breast, gastric, ovarian, bladder and pancreas (Carver et al., 2003). However, our study showed that HSPB1 is downregulated in AML. In addition, the HR of univariate analysis indicated that HSPB1 is a negative factor in AML. The result revealed that the higher the gene expression, the higher the risk of patients. A study demonstrated that HSPB1 is a negative regulator of ferroptotic cancer cell death (Yang et al., 2014). Phosphorylated HSPB1 can not only inhibit apoptosis and induce autophagy (Yu et al., 2012; Yang and Stockwell, 2016), but also reduce cellular iron uptake and lipid ROS production (Yang et al., 2014). It is of great significance for us to study its treatment for ferroptosis-mediated cancer.

Currently, some studies have found ferroptosis-related gene signatures which can predict prognosis genes that can predict AML (Yu et al., 2015). Huang et al. (Yu et al., 2015) developed a 12 FRG-based prognostic risk model comprised of 10 risk-related genes (GPX4, CD44, FH, CISD1, SESN2, LPCAT3, AIFM2, ACSL5, HSPB1, and SOCS1) and 2 protective genes (ACSL6 and G3BP1) to predict clinical outcomes. The 12 FRGs are divided into 4 categories according to their functions: lipid metabolism (GPX4, LPCAT3, ACSL5, ACSL6), antioxidant (CD44, SESN2, AIFM2), iron metabolism (CISD1, HSPB1), and cancer metabolism (SOCS1, FH, G3BP1). Although their prediction value is better than our result by comparing ROC, it is easy to exclude some prognostic genes of AML by using a single marker to construct a prognostic model. In our study, two genes are CNV-driven ferroptosis-related genes which were obtained by CNV and ferroptosis binding analysis. The prognostic model composed of these two genes is more conducive to clinical analysis and judgement.

Recently, some scholars have studied AML and found that atorvastatin has activity on AML by up regulating comprehensive stress pathway and inhibiting oxidative phosphorylation (Yusuf et al., 2020). This study showed that atorvastatin inhibited the oxygen consumption rate of AML cells, which has specific significance for chemotherapy-resistant AML primordial cells dependent on oxidative phosphorylation. This study found that HSPB1 gene is enriched in the pathway of oxidative stress. The expression of HSPB1 gene can be considered to have an impact on the progression of AML. It can be speculated that there may be a compensation mechanism between oxidative phosphorylation of HSPB1 and atorvastatin quinone. In addition, studies have shown that leukemia cells rely on aldhyde dehydrogenase 3a2 (aldh3a2) enzyme to oxidize long-chain fatty aldehydes to prevent cell oxidative damage (Zhang et al., 2015), but do not rely on normal myeloid cell counterparts. At the same time, aldehyde is a by-product of oxidative phosphorylation and increased nucleotide synthesis in cancer. It is produced by lipid peroxide and is the basis of non-caspase dependent cell death and iron death. At present, the dependence of leukemia cells on aldh3a2 has been observed in a variety of mouse and human myeloid leukemia. In addition, the inhibition of aldh3a2 and GPx4 has comprehensive lethality. GPx4 inhibits lipid ROS and then block the ferroptosis process. GPx4 inhibition is a known trigger of iron death, but GPx4 inhibition itself has little effect on AML cells. Inhibition of aldh3a2 provides a therapeutic opportunity for the unique metabolic state of AML cells, and may become a new idea for the treatment of AML in the future.

HSPB1 gene is enriched in multiple oxidative stress pathways, and it is an iron death driving gene. It can be speculated that GPx4 inhibition and HSPB1 gene expression are related to the development of AML. Studies have shown that DNAJB6 can promote the iron death process of ESCC (Tang et al., 2011). In ESCC, the overexpression of DNAJB6 is accompanied by a significant decrease in GPx4 protein level. The study also shows that DNAJB6 plays an anticancer role in the process of ESCC through iron death mechanism. This study also shows that DNAJB6 is a protective factor of AML. In addition, a study on bone marrow damage (Zhou and Chen, 2021)showed that Fanconi anemia complement group D2 (FANCD2), a nuclear protein involved in DNA damage repair, can prevent iron apoptosis mediated damage in bone marrow stromal cells (BMSC). Knockout of FANCD2 increases biochemical events related to iron death (e.g., ferrous accumulation, glutathione consumption and malondialdehyde production). Mechanistically, FANCD2 is involved in regulating the expression of genes and/or proteins of iron metabolism (such as fth1, TF, TFRC, HAMP, HSPB1, slc40a1 and steam3) and lipid peroxidation (such as GPx4). However, progressive BMF in FA patients is closely related to AML. Therefore, the mechanism of the linkage of HSPB1, GPx4 and FANCD2 in AML related diseases needs to be further explored.

This study also has some limitations. Our prognostic model was constructed by existing public datasets, although it is validated by PCR expression test, more prospective investigations are needed to validate its predictive power. In the future, we should continue to pay attention to the role of these two genes in AML pathology by experimental and clinical studies. We believe that our prognostic model based on CNV-driven FRGs is of great significance in predicting the survival of AML patients and will offer novel insight for AML research.

## Data Availability

The original contributions presented in the study are included in the article/Supplementary Material, further inquiries can be directed to the corresponding authors.

## Supplementary Material

Supplementary Figure S1 Clinical significance of the prognostic signature in TCGA set. Risk score in the different ages **(A)** and gender **(B)** *P*-values were calculated by the Wilcoxon test.
